# Multidrug-resistant Aeromonas bacteria prevalence in Nile tilapia broodstock

**DOI:** 10.1186/s12866-023-02827-8

**Published:** 2023-03-24

**Authors:** Ahmed H. Sherif, Amina S. Kassab

**Affiliations:** Fish Disease Department, Animal Health Research Institute AHRI, Agriculture Research Center ARC, Kafrelsheikh, Egypt

**Keywords:** Nile tilapia; Multidrug resistant; Aeromonas bacteria; Hatcheries

## Abstract

**Background:**

*Aeromonas hydrophila* is an opportunistic pathogen. Thus, it has received significant attention mainly in the fish sectors with high production scales. Nile tilapia broodstock confined in the environment of fish hatcheries can be stressed. Hence, they are vulnerable to *A. hydrophila.*

**Results:**

Sequencing of the gyr B gene revealed the presence of 18 different *A. hydrophila* strains (kdy 10,620–10,637), which were deposited in the NCBI under accession numbers ON745861–ON745878. The median lethal doses of the isolates ranged from 2.62 × 10^4^ to 3.02 × 10^6^ CFU/mL. Antibiotic resistant genes, sulfonamide (sul1) and tetracycline (tetA) were found in the eighteen isolates*.* Approximately 83.3% of *A. hydrophila* strains were sensitive to ciprofloxacin and florfenicol. Further, eight *A. hydrophila* strains had high MDR indices at 0.27–0.45. All isolates presented with hemolysin activity. However, only 72.22% of them had proteolytic activity, and only 61.11% could form biofilms. Bacterial isolates harbored different pattern virulence genes, the heat-stable cytotonic enterotoxin (ast), cytotoxic enterotoxin (act), and hemolysin (hly) genes were the most prevalent. Also, a trial to inhibit bacterial growth was conducted using titanium dioxide nanoparticles (TiO_2_ NPs) with three sizes (13, 32, and 123 nm). If *A. hydrophila* strains with a high MDR index were tested against TiO_2_ NPs (20 µg/mL) for 1, 12, and 24 h, those with a small size had a greater bactericidal action than large ones. Bacterial strains were inhibited at different percentages in response to TiO_2_ NP treatment.

**Conclusions:**

Nile tilapia broodstock, mortality is associated with different *A. hydrophila strains*, which harbored virulent and MDR genes. Furthermore, TiO_2_ NPs had bactericidal activity, thereby resulting in a considerable reduction in bacterial load.

**Supplementary Information:**

The online version contains supplementary material available at 10.1186/s12866-023-02827-8.

## Background

Globally, the growing demand for fish and fish products caused by the rapid population growth and the increased preference for the consumption of healthier foods. Nile tilapia (*Oreochromis niloticus*) is one of the most produced species in freshwater aquaculture in several countries worldwide, Egypt is among the countries with a high production rate, and it ranks third in Nile tilapia aquaculture [1].

*Aeromonas* species are ubiquitous gram-negative, rod-shaped microbes that are commonly found in freshwater and estuary environments [2]. Different aquatic ecosystems are inhabited by a wide range of *Aeromonas* spp., these species can be isolated from water, soil, and food, such as meat, ham, raw milk, offal, sausage, vegetables, poultry, fish and shellfish [3, 4]. Further, they are normal inhabitants of the gastrointestinal tract of fish [5]. Due to excessive stocking density and poor farming management, farmed freshwater fish are more vulnerable to outbreaks of motile aeromonas septicemia. The pathogenesis of *Aeromonas* is attributed to the different genes that encode a wide range of virulence factors responsible for disease development in the target host. The common virulence factors of pathogenic *Aeromonas* spp. include three different enterotoxins (act, alt, and ast), hemolysin (hlyA), aerolysin (aerA), flagella (fla), lipase (lip), and elastase (ela) [6]. *Aeromonas* produces different toxins, such as hemolysin, aerolysin, and cytotonic enterotoxins, which are harmful to its hosts [7, 8]. *Aeromonas hydrophila* infection is one of the most devastating bacterial infections, accounting for millions of dollars in losses in the global freshwater aquaculture sector [9, 10]. Antibiotic is a key-stone in bacterial disease control. Consequently, there is a global issue regarding multidrug resistance (MDR), even the World Health Organization (WHO) named 2011 as the year of antibiotic resistance [11]. However, Chilean scallop *Argopecten purpuratus* larvae are produced under hatchery-controlled conditions, they are affected by bacterial diseases outbreaks [12, 13]. Treatment with antibiotics, such as chloramphenicol, which was substituted with florfenicol in *A. purpuratus* production, is associated with an increased risk of developing antibiotic resistance [14]. The mechanism or function of antibiotic resistance in pathogenic bacteria should be determined to choose the best option for treatment [15]. Five common mechanisms of antibiotic resistance for Gram-negative and Gram-positive bacteria are enzymatic hydrolysis, enzymatic modifications of antibiotics by group transfer and redox process, modifications of antibiotic targets, reduced permeability to antibiotics by modifications of porins, and active extrusion of antibiotics by membrane efflux pumps [16]. Egypt is among the countries where antimicrobials are regularly used in aquaculture without veterinarian prescriptions [17]. Furthermore, some antimicrobials are used irresponsibly as growth promotors and as preventive treatments to reduce the incidence of diseases in fish farming [18, 19]. In turn, *Aeromonas* develops an adaptive response to respective antibiotics [20]. Antibiotic resistance is transferred via plasmids, integrons, prophages, and transposons, which can carry virulence genes facilitating the development of antibiotic resistance among *Aeromonas* with multiple virulence genes [19, 21, 22]. *Aeromona*s spp. is resistant to several types of antibiotics, which pose a hazard to human health since these isolates can spread to people via the food chain or direct contact with the aquatic environment [23, 24].

Environmental factors, such as metal availability, salinity, dissolved oxygen, pH, and temperature, and potentially bad management (malnutrition, overfeeding, and overcrowding) in hatchery facilities can cause stress among aquatic animals. Thus, they become more vulnerable to infectious diseases. In contrast, natural disease outbreaks are seldom observed in wild aquatic species because they normally coexist with pathogens until there are significant environmental changes [25]. Moreover, disinfections are regularly used in hatcheries affecting microbial balance, thereby restricting the opportunistic ability of infections to be controlled by natural biological processes [26]. Research on more eco-friendly methods of disease management has been based on the growing political and environmental pressure that can limit the use of antibiotics and other therapeutic agents in aquaculture [27].

The exposure to nano metals resulted in bacterial cells deaths which were due to bioaccumulation within the cell membrane [28–30]. Bacterial deaths were proportionally related to nano metals concentration, size, and exposure time as 50 nm or less may pass through the bacteria cell wall if given enough time [31–34]. Titanium dioxide nanoparticles (TiO_2_ NPs) were prepared and used as antibacterial agents for eliminating human pathogens, such as gram-negative and gram-positive bacteria, and as antifungal agents [35]. Among the different metal oxide nanoparticles, TiO_2_ NPs are economical, stable, and safe for people and the environment [36]. The Food and Drug Administration has recommended the use of TiO_2_ in human nutrition [37].

The current study aimed to evaluate the failure of antibiotic treatments against pathogenic *A. hydrophila* in Nile tilapia to eliminate some highly pathogenic strains of isolated strains using TiO_2_ NPs.

## Materials and methods

### Fish hatcheries

Six tilapia fish hatcheries (TH1-6) are in Kafrelsheikh governorate, Egypt. During the normal hatching production process, moribund broodstock fish with clinical signs were collected and aseptically transferred in transporting clear bags with dechlorinated water. The bags were then placed in containers supplied with ice. The samples were transported to the bacteriology laboratory (Animal Health Research Institute) within 1–2 h for further examination using a tranquilizer (MS-222) and antiseptic iodine, according to a previous study [38].

### Clinical examination

Upon the arrival of the samples, the fish were examined using the standard protocols for the assessment of external and internal pathological lesions based on a previous study [39].

### Bacteriological examination

*A. hydrophila* isolation swab samples were collected from the kidney, liver, spleen, and brain of *O. niloticus*, inoculated into tryptic soy broth (Difco, Detroit, USA), and incubated at 30 °C for 24 h. Then, the inoculum was spread onto *Aeromonas* agar and then incubated at 30 °C for 24 h. Representative colonies that were selected randomly were purified by subculturing onto tryptic soy agar under the same conditions. The isolates were stored at − 80 °C in TS broth with glycerol for further analysis.

### Biochemical profiles

The phenotypic and biochemical features of bacterial isolates were validated according to a previous study [40].

- a. Biochemical tests were performed in triplicate using API20 E based on the manufacturer’s instructions (BioM*é*rieux, Marcy l' Etoile, France).
- b. Biofilm production was investigated using the tube adherence method, according to the study of Christensen et al. [41]. Briefly, bacterial isolates were incubated in plastic conical falcon tubes with TSB at 30 °C for 48 h. The tubes were then emptied, dyed with 0.1% solution of safranin, rinsed with distilled water, and dried. Coating of stained material stuck to the tube revealed biofilm formation.
- c. Hemolytic activity was evaluated by streaking bacterial strains onto tryptic soy agar (Oxoid™) plates supplemented with 5% sheep erythrocytes and incubated for 24 h at 30 °C based on the study of Chen and Huang [42]. The appearance of a clear lytic zone on the surface of agar plates indicated a positive result.
- d. According to the study of Arai et al. [43], proteolytic activity was evaluated by plating bacterial culture onto Brain Heart Infusion Agar (HiMedia) supplemented with 1% fresh egg yolk and incubated at 30 °C for 48 h. The evident proteolytic zone surrounding the cells was used to identify proteolytic activity.

### Partial sequences of the *gyrB* gene

The *Aeromonas* strains were streaked onto Brain Heart Infusion Agar (Difco, the USA) and aerobically incubated at 28 °C for 24 h. The genomic DNA of *Aeromonas* isolates was extracted using the DNA extraction kit (DNeasy Kit, Qiagen, the USA), based on the manufacturer’s protocol. To validate the identity of *Aeromonas* spp., a genus-specific primer pair (*gyrB* F: 5′-TCCGGCGGTCTGCACGGCGT-3′ and R 5′-TTGTCCGGGTTGTACTCGTC-3′) was used in the polymerase chain reaction (PCR) amplification [44]. Briefly, a reaction mixture containing 12.5 μl of Dream Taq Green PCR Mix (2X) (Thermo-Scientific, the USA), 2 µl of extracted DNA, 1 µl of each primer, and 8.5 µl of distilled water. The thermal cycler program was adjusted as follows: 95 °C for 4 min (initial denaturation), followed by 30 cycles of denaturation at 95 °C for 30 s, annealing at 55 °C for 30 s, and extension at 72 °C for 90 s. The reaction was ended at 72 °C for 10 min (as the final extension). The amplicons were purified using the A GeneJET™ PCR Purification Kit (Thermo Fisher Scientific, the USA). PCR products were electrophoresed in 1.5% agarose and visualized under ultraviolet light.

The amplified *gyrB* gene was sequenced in two directions using the ABI 3730xl DNA sequencer (Applied Biosystems, the USA). The raw sequences were edited and assembled using BioEdit version 7.0 [45]. The assembled *gyrB* genes were submitted to GenBank. The phylogenetic tree was constructed using MEGA version X [46]. Neighbor-joining phylogenetic analysis was performed using the Kimura two-step algorithm with 1,000 bootstrap replicates.

### Examination of virulence and antibiotic-resistant genes

The virulence genes were cytotoxic enterotoxin (act), heat-stable cytotonic enterotoxin (ast), cytotoxic enterotoxin (alt), hly, serine protease (ser), and extracellular lipase (lip). Some antibiotic resistant genes namely sul1, tetA, quinolone (qnrs), and erythromycin (ermB) were screened in all *Aeromonas* isolates using PCR, as described in the study of Randall et al. [47]. All primers used in this work are inserted in (Table 1).

**Table 1:** Primers used to detect bacteria and antibiotic-resistant genes

Primer	Sequence	Annealing temperature (°C)	Product size (pb)	References
Act	F: AGAAGGTGACCACCACCAAGAACA R: AACTGACATCGGCCTTGAACTC	55	232	[21]
Ast	F: TCTCCATGCTTCCCTTCCACT R: GTGTAGGGATTGAAGAAGCCG	55	331	
Alt	F: TGACCCAGTCCTGGCACGGC R: GGTGATCGATCACCACCAGC	55	442	
Ser	F: CACCGAAGTATTGGGTCAGG R: GGCTCATGCGTAACTCTGGT	50	350	
Lip	F: CAYCTGGTKCCGCTCAAG R: GTRCCGAACCAGTCGGAGAA	58	247	
Hly	F: GGCCGGTGGCCCGAAGATACGGG R: GGCGGCGCCGGACGAGACGGGG	55	592	[48]
SulI	F: CGGCGTGGGCTACCTGAACG R: GCCGATCGCGTGAAGTTCCG	68	433	[49]
TetA	F: GCTACATCCTGCTTGCCTTC R: CATAGATCGCCGTGAAGAGG	55	210	[50]
qnrs	F: ACGACATTCGTCAACTGCAA R: TAAATTGGCACCCTGTAGGC	53	417	[51]
ermB	F: TGGTATTCCAAATGCGTAATG R: CTGTGGTATGGCGGGTAAGT	62	745	[52]

### Antibiogram of *Aeromonas* strains

The disc diffusion method was used to assess the antibiotic susceptibility of bacterial isolates using Mueller–Hinton agar (Oxoid™) based on a previous study [53]. The antimicrobial agents tested were tetA 30 μg, trimethoprim/sulfamethoxazole (SXT) 1.25/23.75 μg), ciprofloxacin (CIP) 5 μg, florfenicol 30 μg, erythromycin (E, 15 μg), gentamicin 10 μg, amoxicillin 10 μg, ampicillin (AMP) 10 μg, kanamycin 30 μg, cefotaxime 30 μg, and streptomycin 30 μg). The test was performed in triplicates. Isolates were subcultured in tryptic soy broth, incubated overnight at 30 °C, and streaked onto Mueller–Hinton agar plates using a cotton swab. The antibiotic discs were adjusted on the agar surface and incubated for 24 h at 30 °C. The diameter of the inhibition zones was determined, and the results were interpreted according to the criteria of the Clinical Laboratory Standards Institute,.MDR index was calculated as X*/*Y, where X is all types of antibiotics wherein the isolates were resistant to and Y is all antibiotics used in the study. An MDR index of > 0.2 indicated resistance to multiple antibiotics [54].

### Median lethal dose (LD_50_)

The median lethal dose (LD_50_) of *A. hydrophila* was evaluated according to the procedure of Reed & Muench [55]. Briefly, *O. niloticus* (40 ± 3 g b.w.) was acclimated at the wet laboratory of the Animal Health Research Institute. After anesthetizing the fish using tricaine methanesulfonate (MS222; Sigma, St. Louis, MO, the USA), groups of 10 fish were intraperitoneally injected with serial tenfold dilutions of *A. hydrophila* cultured in Brain Heart Infusion Broth at 30 °C for 24 h. First, 100 μl of *A. hydrophila* suspension was adjusted to 1 × 10^2^, 1 × 10^3^, 1 × 10^4^, 1 × 10^5^, 1 × 10^6^, 1 × 10^7^, 1 × 10^8^, 1 × 10^9^, or 1 × 10^10^ CFU/mL in normal saline (0.65%). The suspension was injected into duplicate groups of five fish. The 14-day mortality rates were recorded, and *A. hydrophila* was re-isolated from the dead moribund fish and confirmed via PCR. The clinical signs were recorded and photographed.

### Manufacturing and impact of titanium dioxide on bacterial cells

Titanium (IV) isopropoxide (TIP) (C12H28O4Ti) (purity: 97%), 2-propanol [(CH3)2CHOH] (purity: 99%), and hydrochloric acid (HCL) (concentration: 98%) were purchased from Sigma-Aldrich. A mixture of TIP and 2-propanol with a ratio of 1:4 was stirred using a magnetic stirrer at 200 rpm for 1 h at room temperature. Another mixture of deionized water and 2-propanol with a ratio of 1:1 was added to the first one drop by drop. Then, the mixture was stirred as above. The pH value of the solution was adjusted at pH = 3, 3.5, or 4 using HCL then final solution was stirred as above. The final mixture was placed in a water bath at 80 °C to evaporate any liquid in the mixture. The resulting white powder TiO_2_ was hydrogenated for at different temperatures (300 °C, 400 °C, 500 °C, and 600 °C) using (tubular furnace). Three different nanosized particles of TiO_2_ anatase 13, 32, and 123 nm were formulated. Then, the shapes were photographed via a JEOL expository scanning electron microscope (SEM) device [56].

The time-dependent antibacterial activity of TiO_2_ NPs was evaluated as follows: Briefly, 50 µl of 24-h old *A. hydrophila* inoculum (corresponding to a concentration of the calculated LD_50_ of the MDR strains) was exposed to a dilution of the three sized-TiO_2_ NPs 20 µg/mL for 1, 12, and 24 h, according to previous studies [30, 33, 57]. The culture grew in triplicate on standard count plate (Sigma-Addrich) at 37 °C for 24 h. Meanwhile, the tryptic soya broth alone was considered as the negative control. The bacteria counts were calculated by the number of growing colonies in the culture media.

### Biosafety procedure

This study followed the biosafety measures on the pathogen safety data sheets (Infectious substances–*A. hydrophila*, Pathogen Regulation Directorate [58].

## Results

### Genetic identification of *Aeromonas* spp.

The *gyrB* gene was successfully amplified from all 18 isolates of *Aeromonas* spp. (kdy 10,620–kdy 10,637). The multiple alignments of the *gyrB* gene sequences confirmed that all 18 strains belonged to the genus *Aeromonas*. The accession numbers obtained from the sequencing of the *gyrB* genes ranged from ON745861 to ON745878. The BLAST analysis of these sequences showed a 99.91%–97.06% similarity to *A. hydrophila* (AB436660^T^, AB436661^T^, AY987520^T^, CP000462^T^, AJ868394^T^, OL321923, OL321922, and AB473068). Consequently, the current *Aeromonas* strains were genetically identified as *A. hydrophila*. The intraspecies similarity of *A. hydrophila* isolates was 99.81%–100% with 1–2-bp nucleotide differences.

**Fig. 1:**
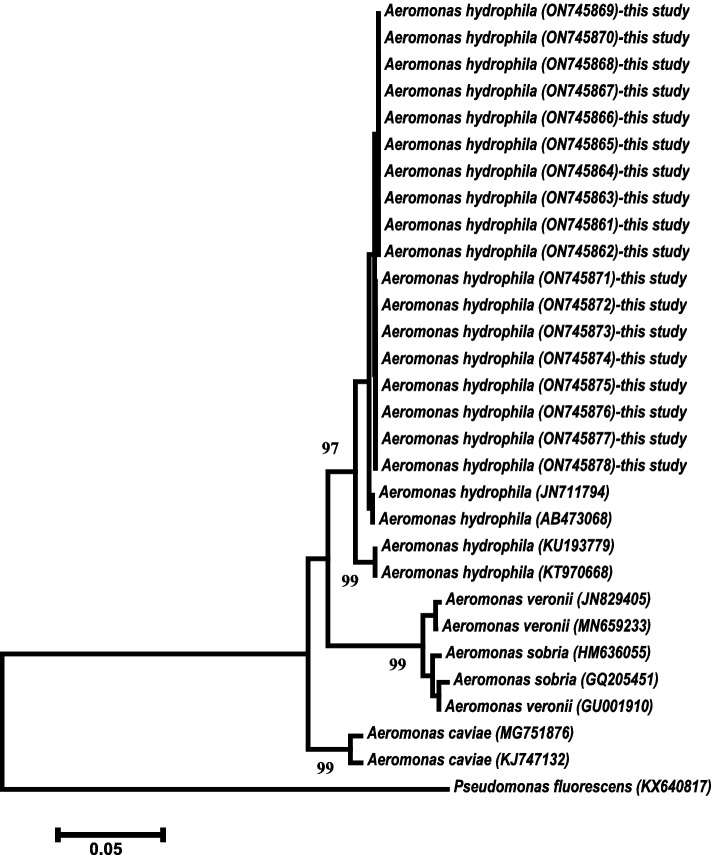
Phylogenetic tree of the isolates in this study

By contrast, the neighbor-joining phylogenetic analysis showed two large clades. The first lineage was further divided into two subclades. The first one clustered the current 18 strains of *A. hydrophila* with other *A. hydrophila* isolates recovered from GenBank with a bootstrap value of 97% and separated from the second subclade. Then, the second subclade clustered *A. veronii* isolates with *A. sobria* isolates to form one subclade with a high bootstrap value at 99%. Further, it included *A. caviae* isolates with a high bootstrap value of 99% (Fig. 1). *Pseudomonas fluorescens* (KX640817) was used as an outgroup isolate.

### Detection of MDR and ARG in the isolated strains

In Table 2, the MDR index was between 0.27 and 0.45 in eight *A. hydrophila* strains (ON745861, ON745862, ON745863, ON745866, ON745867, ON745876, ON745877, and ON745878). Meanwhile, it was 0.18 in 10 strains.

**Table 2:** Data on bacterial strains, accession number, site, virulence genes, and multidrug resistant genes

**Bacterial strain**	**Accession number**	**Site**	**MDR**	**Antibiotic**	**ARG**
kdy 10,620	ON745861	TH1	0.45	TE, SXT, CIP, E, AMP	Sul1, tetA, qnrs
kdy 10,621	ON745862	TH1	0.45	TE, SXT, CIP, E, AMP	Sul1, tetA, qnrs
kdy 10,622	ON745863	TH1	0.36	TE, SXT, CIP, FFC	Sul1, tetA, qnrs
kdy 10,623	ON745864	TH2	0.18	TE, SXT,	Sul1, tetA
kdy 10,624	ON745865	TH2	0.18	TE, SXT,	Sul1, tetA
kdy 10,625	ON745866	TH3	0.27	TE, SXT, FFC	Sul1, tetA, qnrs, ermB
kdy 10,626	ON745867	TH3	0.27	TE, SXT, FFC	Sul1, tetA, qnrs, ermB
kdy 10,627	ON745868	TH3	0.18	TE, SXT	Sul1, tetA, qnrs, ermB
kdy 10,628	ON745869	TH4	0.18	TE, SXT	Sul1, tetA, ermB
kdy 10,629	ON745870	TH4	0.18	TE, SXT	Sul1, tetA, ermB
kdy 10,630	ON745871	TH5	0.18	TE, SXT	Sul1, tetA
kdy 10,631	ON745872	TH5	0.18	TE, SXT	Sul1, tetA
kdy 10,632	ON745873	TH5	0.18	TE, SXT	Sul1, tetA
kdy 10,633	ON745874	TH5	0.18	TE, SXT	Sul1, tetA
kdy 10,634	ON745875	TH5	0.18	TE, SXT	Sul1, tetA
kdy 10,635	ON745876	TH6	0.27	TE, SXT, E	Sul1, tetA, qnrs
kdy 10,636	ON745877	TH6	0.27	TE, SXT, E	Sul1, tetA, qnrs
kdy 10,637	ON745878	TH6	0.36	TE, SXT, E, AMP	Sul1, tetA, qnrs

*MDR* multidrug resistant genes, *TE* tetracycline, *SXT* trimethoprim/sulfamethoxazole, *CIP* ciprofloxacin, *FFC* florfenicol, *E* erythromycin, *AMP* ampicillin; sul1, sulfonamide, *tetA* tetracycline, *qnrs* quinolone; and ermB, erythromycin

The antibiotic resistance genes sul1 and tetA were found in 100% (18/18) of the isolates, qnrs in 44.4% (8/18), and ermB in 27.78% (5/18).

### Phenotypic antibiotic resistance

Table 3 shows the bacterial strains (kdy 10,620–kdy 10,637), all bacterial strains were resistant to tetracycline, trimethoprim & sulfamethoxazole. Meanwhile, 83.3% of bacterial strains were sensitive to ciprofloxacin and florfenicol and 72.23% to erythromycin. In addition, 33.33%, 27.77%, and 38.9% of isolates were less sensitive to gentamycin, amoxicillin, and ampicillin, respectively.

**Table 3:** Antibiogram of the isolated bacteria

**Antibiotic (8)**		***A. hydrophila***	**%** *n* = 18
**Tetracycline** 30 μg	S	0	0
	IM	0	0
	R	18	100
**Trimethoprim** 1.25 μg **Sulfamethoxazole** 23.75 μg	S	0	0
	IM	0	0
	R	18	100
**Ciprofloxacin** 5 μg	S	15	83.33
	IM	0	0
	R	3	16.67
**Florfenicol** 30 μg	S	15	83.33
	IM	0	0
	R	3	16.67
**Erythromycin** 15 μg	S	13	72.23
	IM	0	0
	R	5	27.77
**Gentamycin** 10 μg	S	6	33.33
	IM	12	66.67
	R	0	0
**Amoxicillin** 30 μg	S	5	27.77
	IM	13	72.23
	R	0	0
**Ampicillin** 10 μg	S	7	38.9
	IM	8	61.1
	R	3	16.67
**Kanamycin** 30 μg	S	10	55.56
	IM	8	44.4
	R	0	0
**Cefotaxime** 30 μg	S	9	50
	IM	9	50
	R	0	0
**Streptomycin** 30 μg	S	12	66.67
	IM	6	33.33
	R	0	0

*S* sensitive, *IM* intermediate, *R* resistant

### Virulence genes and biochemical identification of *Aeromonas* spp.

As shown in Table 4, the virulence genes heat-stable cytotonic enterotoxin (ast), cytotoxic enterotoxin (act, alt), hemolysin (hly), serine protease (ser), and extracellular lipase (lip) were detected in 18 isolates at different percentages (83.3% [15/18], 100% [18/18], 44.4% [8/18], 100% [18/18], 50% [9/18], and 50% [9/18], respectively) in bacterial strains kdy 10,620–10,638.

**Table 4:** Virulence genes and biochemical characteristics of the bacterial isolates

**Bacterial strain**^a^	**Virulence Genes**	**Biofilm production**	**Hemolysin activity**	**Proteolytic Activity**
kdy 10,620	ast, act, alt, hly, ser, lip	** + **	** + **	** + **
kdy 10,621	act, alt, hly, ser, lip	** + **	** + **	** + **
kdy 10,622	act, alt, hly, ser, lip	** + **	** + **	** + **
kdy 10,623	act, hly, ser, lip	**-**	** + **	** + **
kdy 10,624	ast, act, hly	**-**	** + **	**-**
kdy 10,625	ast, act, alt, hly, ser, lip	** + **	** + **	** + **
kdy 10,626	ast, act, alt, hly, ser, lip	** + **	** + **	** + **
kdy 10,627	ast, act, hly	** + **	** + **	**-**
kdy 10,628	ast, act, hly	** + **	** + **	**-**
kdy 10,629	ast, act, hly	** + **	** + **	**-**
kdy 10,630	ast, act, hly	**-**	** + **	**-**
kdy 10,631	ast, act, hly	**-**	** + **	**-**
kdy 10,632	ast, act, hly	**-**	** + **	**-**
kdy 10,633	ast, act, hly	**-**	** + **	**-**
kdy 10,634	ast, act, hly	**-**	** + **	**-**
kdy 10,635	ast, act, alt, hly, ser, lip	** + **	** + **	** + **
kdy 10,636	ast, act, alt, hly, ser, lip	** + **	** + **	** + **
kdy 10,637	ast, act, alt, hly, ser, lip	** + **	** + **	** + **

^a^Virulence genes: ast, heat-stable cytotonic enterotoxins; act, cytotoxic enterotoxin; alt, cytotoxic enterotoxin; hly, hemolysin; ser, serine protease; and lip, lipase

Bacterial isolates possess different biochemical characters under identification numbers 107126, 7,456,754, 7,467,754, and 7,576,755 using API20E. Also, biofilm production, hemolysin activity, and proteolytic activity were detected in the bacterial isolates with percentages of 61.11% (11/18), 100% (18/18), and 72.22% (13/18), respectively.

### Bacterial pathogenesis

**Fig. 2:**
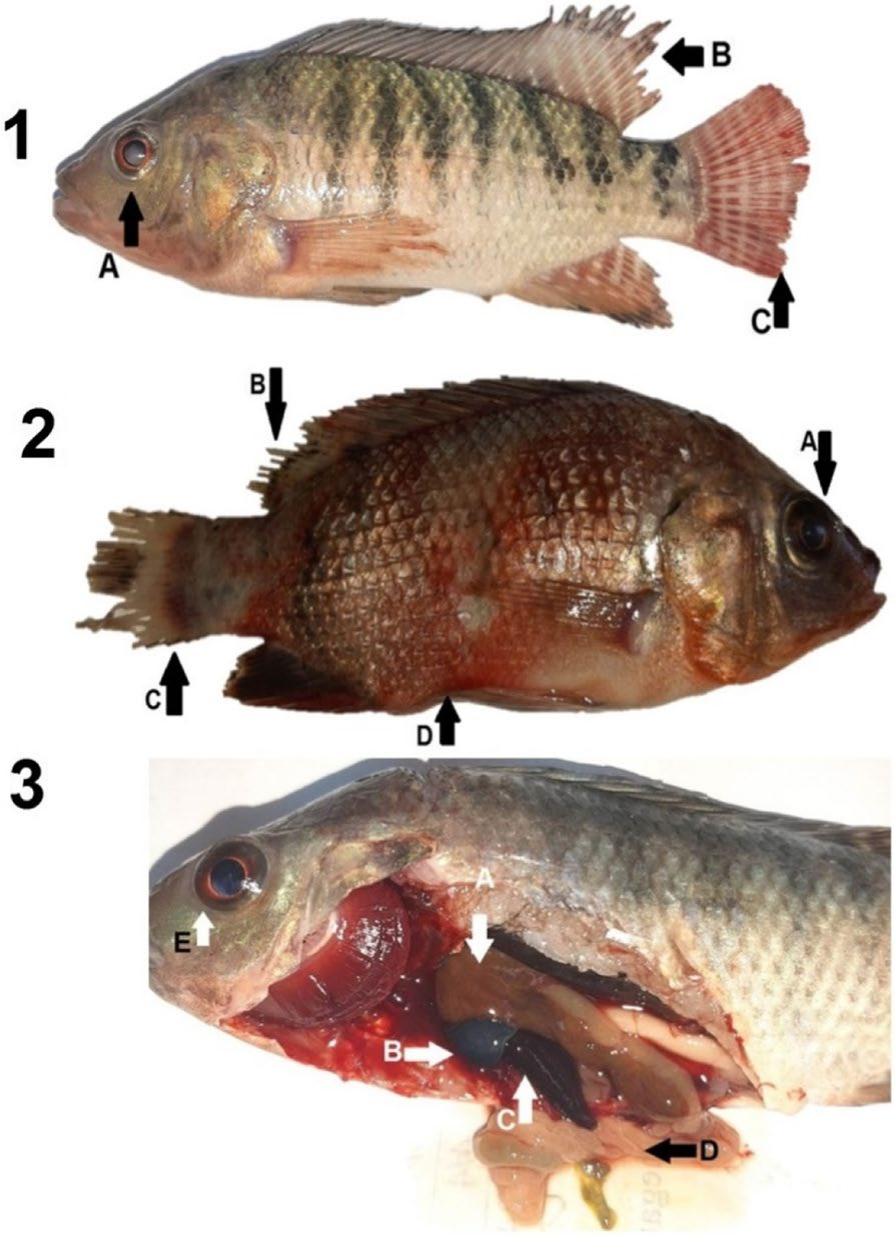
(1) Arrows–A: Opaque, slightly protruded eye. B: Dentated dorsal fin. C: Dentated tail fin. (2) Arrows–A: Opaque eye. B: Extensive dentated dorsal and tail fin. D: Empty abdomen with hemorrhagic body surface and sloughed scales. (3) Arrows–A: Dark brown liver. B: Distended gall bladder. C: Splenomegaly. D: Empty intestinal tract. E: Slightly opaque eye

In Fig. 2, the fish presented with the following clinical signs: opaque, slightly protruded eye, dentated dorsal and tail fin, hemorrhagic body surface, and sloughed scales. The post mortem signs were dark brown liver, distended gall bladder, splenomegaly, and empty intestinal tract.

As depicted in Table 5, the LD_50_s of ON745861, ON745862, ON745863, ON745866, ON745867, ON745876, ON745877, and ON745878 were 1.71 × 10^5^, 1.93 × 10^5^, 1.00 × 10^5^, 2.37 × 10^6^, 3.02 × 10^6^, 1.43 × 10^6^, 2.62 × 10^4^, and 1.92 × 10^5^ CFU/mL, respectively.

**Table 5:** Effect of TiO_2_ NPs with different sizes on bacterial strains after 24 h of incubation

Bacterial strain	LD_50_ CFU/mL (%)	TiO_2_ NPs (13 nm)			TiO_2_ NPs (32 nm)			TiO_2_ NPs (123 nm)		
		**1 h**	**12 h**	**24 h**	**1 h**	**12 h**	**24 h**	**1 h**	**12 h**	**24 h**
**kdy 10,620**	1.71 × 10^5^ (100)	0.44 × 10^5^ (25.73)	0.4 × 10^3^ (0.23)	0 (0)	0.5 × 10^5^ (29)	1.3 × 10^3^ (0.76)	0 (0)	0.82 × 10^5^ (47.95)	1.4 × 10^3^ (0.99)	0 (0)
**kdy 10,621**	1.93 × 10^5^ (100)	2.1 × 10^3^ (10.88)	0.25 × 10^3^ (0.13)	0 (0)	0.3 × 10^5^ (15.54)	0.77 × 10^3^ (0.4)	0 (0)	1.1 × 10^5^ (57)	1.33 × 10^3^ (0.69)	0 (0)
**kdy 10,622**	1.00 × 10^5^ (100)	0.1 × 10^5^ (10)	0.15 × 10^3^ (0.15)	0 (0)	0.39 × 10^5^ (39)	1.21 × 10^3^ (1.21)	0 (0)	0.42 × 10^5^ (42)	1.49 × 10^3^ (1.49)	0 (0)
**kdy 10,625**	2.37 × 10^6^ (100)	0.92 × 10^6^ (38.82)	0.7 × 10^3^ (0.03)	0 (0)	1.45 × 10^6^ (61.18)	0.73 × 10^4^ (0.31)	0 (0)	1.62 × 10^6^ (68.35)	1.9 × 10^4^ (0.8)	0 (0)
**kdy 10,626**	3.02 × 10^6^ (100)	0.79 × 10^6^ (26.15)	0.02 × 10^4^ (0.007)	0 (0)	0.98 × 10^6^ (32.45)	1.67 × 10^4^ (0.55)	0 (0)	1.84 × 10^6^ (60.9)	1.74 × 10^4^ (0.58)	0 (0)
**kdy 10,635**	1.43 × 10^6^ (100)	0.58 × 10^6^ (40.56)	0.85 × 10^4^ (0.6)	0 (0)	0.66 × 10^6^ (46.15)	1.23 × 10^4^ (0.86)	0 (0)	0.92 × 10^6^ (64.34)	1.34 × 10^4^ (0.94)	0 (0)
**kdy 10,636**	2.62 × 10^4^ (100)	1.13 × 10^4^ (43.13)	0.23 × 10^2^ (0.09)	0 (0)	1.49 × 10^4^ (56.87)	1.36 × 10^2^ (0.52)	0 (0)	1.53 × 10^4^ (58.4)	1.7 × 10^2^ (6.49)	0 (0)
**kdy 10,637**	1.92 × 10^5^ (100)	1.1 × 10^5^ (57.3)	1.32 × 10^2^ (0.07)	0 (0)	0.84 × 10^5^ (43.75)	1.51 × 10^3^ (0.79)	0 (0)	0.98 × 10^5^ (51)	2.1 × 10^3^ (1.09)	0 (0)

*LD*_*50*_ median lethal dose, *CFU* colony forming unit, *TiO*_*2*_* NPs* titanium dioxide nanoparticles

### Antibacterial effect of TiO_2_ NPs

**Fig. 3:**
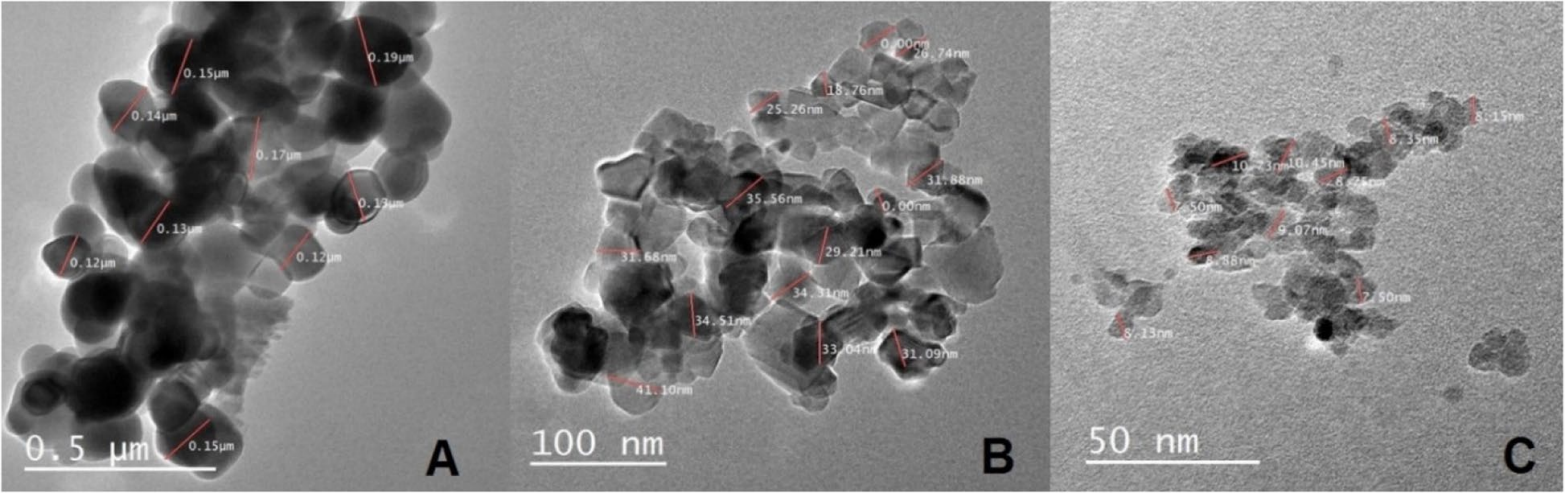
Size and shape of titanium dioxide nanoparticles on scanning electron microscope

Figure 3 shows the characteristics of TiO_2_ NPs. The average sizes of anatase crystalline were 13, 32, and 123 nm. The TiO_2_ NPs (anatase) was nano powder, and its purity was 97.45%. To confirm the TiO_2_ NPs purity, size and shape were evaluated using SEM, as shown in the Supporting Information.

The antibacterial properties of three nanosized TiO_2_ (at a concentration of 20 µg/mL) were evaluated against the highly pathogenic *A. hydrophila* for 1, 12, and 24 h (Table 5). Regardless of bacterial strain and exposure time, the small-size TiO_2_ NPs had a higher bactericidal activity (13 > 32 > 123 nm) than the larger sizes. After 1 h of exposure to TiO_2_ NPs measuring 13 nm, the bacterial count ranged from 10% to 57.3% from the initial count (LD_50_). Meanwhile, TiO_2_ NPs measuring 32 nm had decreased bacterial count at 15.59%–61.18%. Finally, the bacterial count was between 42% and 68.35% after exposure to TiO_2_ NPs measuring 123 nm. After 24 h, there was no bacterial growth. Regardless of TiO_2_ NPs size and exposure time, the bacterial count varied in different bacterial isolates.

## Discussion

*Aeromonas hydrophila* was isolated from moribund broodstock Nile tilapia in different six hatcheries. They were recognized phenotypically and genotypically, and the sequence of *gyrB* gene revealed the presence of 18 *Aeromonas hydrophila* strains. (kdy 10,620–kdy 10,637). During disease outbreaks, the accurate identification of the causal pathogens is a challenge to fish pathologists [59]. Similarly, some researchers found that *Aeromonas* strains could be identified by sequencing *gyrB,* 16 s rRNA*,* and *rpoD* genes [60].

In this study, all *Aeromonas* strains were resistant to tetracycline, trimethoprim & sulfamethoxazole. Meanwhile, 83.3% of the strains were sensitive to ciprofloxacin and florfenicol and 72.23% to erythromycin. Furthermore, 33.33%, 27.77%, and 38.9% of the strains were less sensitive to gentamycin, amoxicillin, and ampicillin, respectively. Similarly, opportunistic pathogenic bacteria can resist a wide range of antibiotics causing bacterial infections, particularly in immune-compromised individuals [61]. Accordingly, aeromonads were sensitive to fluoroquinolones, and ciprofloxacin could be the treatment of choice [2, 62]. Similarly, aeromonads collected from diseased fish were resistant to ampicillin, amoxicillin, and erythromycin [63]. On the contrary, only 15% of *Aeromonas* spp. were resistant to tetracycline [64]]. Furthermore, 7.7%, 17.9%, 25.6%, 43.6%, and 47.7% of *Aeromonas* spp. were resistant to doxycycline, trimethoprim/sulfamethoxazole, chloramphenicol, colistin, and erythromycin [63]. These differences in percentages of resistant bacteria could be due to the date of conducting the survey, the veterinary choice for the antibiotic, and the history of antibiotic abuse in the fish farm.

The MDR indices were high, ranging from 0.27 to 0.45, in eight *A. hydrophila* strains (kdy 10,620, kdy 10,621, kdy 10,622, kdy 10,625, kdy 10,626, kdy 10,635, kdy 10,636, and kdy 10,637). Meanwhile, the index was 0.18 in the remaining isolates in this study. Similarly, Liu et al. [65] found that the MDR in *A. hydrophila* pathogenic strains ranged from 0.2 to 0.6. The MAR index of *A. hydrophila* (87.2%) was > 0.2. Thus, these strains were obtained from a high-risk source of infection [54]. Accordingly, the MDR was 0.387 regardless of the sources of *Aeromonas* strains, which were cultured freshwater animals in China [66]. Meanwhile, the MDR was 0.263 and 0.287 in Aeromonads isolated from human patients [67, 68].

In this investigation, ARG sul1 and tetA were found in all *A. hydrophila* isolates, while ARG qnrs and ermB were in 44.4% and 27.78% of the isolates, respectively. Accordingly, 26.3% of pathogenic bacteria detected in Mediterranean fish farms carried at least one of the tetracycline RGs [69] and tetracycline RGs accounted for more than half of the total ARGs isolated from infected bacteria in fish and ducks [70]. In addition, sulphonamide RGs (sulII and sulIII) were detected in 56.14% of bacterial isolates in fish tanks [71]. Moreover, quinolone RGs (qnrs) were discovered in *Aeromonas* spp. isolated from the Seine River in Paris [72]. Accordingly, Jang et al. [73] recorded a low incidence of ermC-encoding erythromycin-resistant pathogens isolated from the effluent of coastal aquaculture in Jeju Island, South Korea. Similarly, Deng et al. [66] found that at least 18.86% of *Aeromonas* strain isolates from cultured freshwater animals were resistant to trimethoprim/sulfamethoxazole and that 18.9% and 4.7% of them also carried RG for sul1 and qnrs. Similarly, to our findings, Shuang et al. [74] revealed that sul1 and qnrs were present in *Aeromonas* strains at rates of 12.1% and 22.8%, respectively, and that these bacteria were trimethoprim/sulfamethoxazole-resistant.

In this study, the virulence genes were detected in the eighteenth *A. hydrophila* strains (kdy 10,620–10,638) with various percentages of ast (83.3%), act (100%), alt (44.4%), hly (100%), ser (50%), and lip (50%). Similarly, approximately 50% of *A. hydrophila* had 50% of the virulence genes tested (namely, aer, ahp, alt, hly, lip, fla, ela, and/or act) meanwhile, none were found in 2% [64]. Based on our findings, although with different percentages recovered, the virulence genes were found in 41% of *A. hydrophila* isolates, including aer (33.33%), lip (23.1%), hlyA (5.13%), and ast (2.56%) [63]. Accordingly, the lip gene was detected in 50% of *A. hydrophila*, and it could alter the cell membrane structure of fish tissues and, thus, could manifest bacterial pathogenicity [21].

In this study, *A. hydrophila* had more than one virulence gene, and the most common virulence gene patterns were ast, act, and hly. Similarly, the virulence gene *ast* was the most isolated among 94 *Aeromonas* isolates, and additional virulence genes, such as alt, ast, act, aer ser, fla, and hly, were also discovered [74, 75]. Moreover, the most prominent virulence gene pattern was aer/hly/fla*,* with a prevalence rate of 12.6% [76]. In addition, an association was discovered between the lip and aer genes and the hlyA and ast genes in *A. hydrophila* isolated from *Mugil cephalus* indicating a potential synergy among these genes during infection [62]. Certainly, the virulence genes could share genomic locations on mobile genetic elements [77]. Different findings, El-Bahar et al. [78] claimed that hlyA gene, which destroys red blood cells and causes anemia, was detected in 10% of *A. hydrophila* strains. Furthermore, it was not detected among *Aeromonas* strains from freshwater lakes in Malaysia [79]. Moreover, only 5% of *A. hydrophila* isolates harbored the ast gene, which can promote gut vascular permeability and intestinal mucosal detachment [80]. Previous findings could explain the occurrence of Aeromoniasis as virulence factors, either alone or in combination, may allow *Aeromonas* spp. to invade host cells, thereby overlapping the immunological response and causing diseases [81, 82].

The clinical signs in experimental *O. niloticus* infected with *A. hydrophila* are similar to those of septicemic bacteria (opaque, slightly protruded eye, dentated dorsal, and tail fin, hemorrhages on the body surface, and sloughed scales). Meanwhile, the post-mortem signs were dark brown liver, distended gall bladder, splenomegaly, and empty intestinal tract. Similarly, *Aeromonas* infections resulted in exophthalmia, hemorrhage, ulceration, fin rot, lethargy, loss of scale, skin discoloration, and hemorrhagic/necrotized internal organs in different types of fish [83, 84]. The observed clinical signs are attributed to bacterial toxins such as aerolysin and cytotoxic enterotoxin, which can cause damage to body tissues and erythrocyte membranes resulting in hemorrhagic scales; fin and tail atrophy of broodstock Nile tilapia. In this investigation, 61.11% of *A. hydrophila* could produce a biofilm. In accordance, most bacterial species in the aquatic system could form biofilm [85, 86], which increased virulence and resistance that may potentially decrease the LD_50_ by increasing the viable bacterial cells, impervious to ordinary antimicrobial agents and disinfectants, thereby becoming a repository for the spread of pathogenic bacteria [87]. Multiple virulence genes may manage pathogenic *A. hydrophila* to combat normal commensal bacteria [88], and they have synergistic effects on pathogenicity [89] and act in combination with other virulence factors, such as biofilm production, hemolysin activity, and proteolytic activity, which could act synergistically to cause clinical diseases [80].

The antibacterial activity of several metal oxide nanoparticles, such as TiO2, is effective against both gram-negative and gram-positive bacteria [90, 91]. Cell death has been recorded because of nanomaterials aggregation within the bacterial membrane [28]. Based on previous findings on MDR and virulence genes, TiO_2_ NPs were considered in the treatment of the isolated *A. hydrophila,* three TiO_2_ NPs measuring 13, 32, and 123 nm were assessed against the highly pathogenic *A. hydrophila* (gram-negative) in time factor (1, 12, and 24 h).

The growth of *A. hydrophila* was suppressed more effectively and swiftly with small-size TiO2 NPs (13 nm) than with large-size ones. Similar to our findings on different bacterial spp., Ngoepe et al. [92] reported that TiO_2_ with a size of 6–10 nm had a high antibacterial activity at a concentration of 0.05 mg/mL, which was selectively active against gram-negative *E. coli* strain. Similarly, smaller-size NPs had more significant toxicity than larger ones when administered at the same concentration [93, 94]. These findings are attributed to the fact that smaller-sized NPs not only had a larger surface area but could also cross cell membrane barriers and accumulate inside the bacterial cells [29, 95]. Similarly, Sherif et al. [96] showed that TiO_2_ NPs (anatase crystal) could affect the microbiota in Nile tilapia, thereby reducing the number of beneficial bacteria. However, Sherif et al. [97] found that nano selenium could inhibit the recurrence of *A. hydrophila* infection. By contrast, Garcidueñas-Piña et al. [98], TiO_2_-Cu2 + nanoparticles did not exhibit bactericidal action against *E. coli* even at the highest concentration (10 mg/mL).

After 1 h of exposure to TiO_2_ NPs, small-size NPs (13 nm) were more effective than large-size ones (32 and 123 nm). In addition, after 24 h, the growth of *A. hydrophila* was suppressed of regardless the particle size. In previous studies, on exposure to graphene oxide NPs, a positive correlation was observed between cell death and exposure time [99] and concentration [34]. That is, if the cell culture exposed to nanoparticles was longer, the cell death was higher [99]. Accordingly, Rincon & Pulgarin [100] confirmed that a longer exposure duration is required for bacterial inactivation if the initial concentration of bacteria is higher. Whereas, other studies showed that TiO_2_ needs more time to exert its action, as the percentage of cell death was 75% after 96 h of incubation [57].

## Conclusion

Mortality in Nile tilapia broodstock is attributed to stress conditions. Hence, these animals were vulnerable to 18 isolates of *A. hydrophila* harboring different patterns of virulence genes as the heat-stable cytotonic enterotoxin (ast), cytotoxic enterotoxin (act), and hly genes were the most prevalent. The MAR index ranged from 0.27 to 0.45 in eight *A. hydrophila* strains. Meanwhile, it was 0.18 in the other strains. The resistant genes sul1 and tetA were found in 100% of the bacterial isolates, while qnrs and ermB were present in 44.4% and 27.78%. Furthermore, TiO_2_ NPs had bactericidal activity, thereby resulting in a considerable reduction in bacterial load, it was noticed that the lower the nanosize the lower the bacterial count as the lowest bacterial count (10% to 57.3%) after 1 h of the exposure to TiO_2_ NPs measuring 13 nm.

## Supplementary Information

**Additional file 1.**

## Data Availability

Data are available on request from the corresponding author.
