# Supplementary material for: Multidrug-resistant Aeromonas bacteria prevalence in Nile tilapia broodstock
Source: BMC Microbiol. 2023 Mar 24;23:80. doi: 10.1186/s12866-023-02827-8 (PMC10037768; doi:10.1186/s12866-023-02827-8)
Supplement: Supplementary file 1 — Additional file 1. [file 12866_2023_2827_MOESM1_ESM.pdf]

Author First, Quality First
www.enago.com

localize to globalize
www.ulatus.com

we are all ears
www.voxtab.com

CERTIFICATE OF EDITING

This is to certify that the paper titled Prevalence of multidrug resistance in Aeromonas bacteria in Nile tilapia broodstock commissioned to us by Amina kassab has been edited for English language, grammar, punctuation, and spelling by Enago, the editing brand of Crimson Interactive Inc. under Copyediting/Language editing.

- **ISO 17100:2015** Translation Service Providers
- **ISO 27001:2013** Information Security Management System
- **ISO 9001:2015** Quality Management System

Issued by:

Enago, Crimson Interactive Inc.
160, Greentree Dr, Ste 101 street,
Dover City, Kent, Delaware, 19904
Phone: +1-302-498-8358

Disclaimer : The intent of the author's message has been preserved during the editing process. The author is free to accept or reject our changes in the document after reviewing our edits. This certificate has been awarded at the time of sharing the final edited version (full file or sections of the file) with the author. Enago does not bear any responsibility for any alterations done by the author to the edited document post 28 Nov 2022.

**Japan**	www.enago.jp, www.ulatus.jp, www.voxtab.jp
**Taiwan**	www.enago.tw, www.ulatus.tw
**China**	www.enago.cn, www.ulatus.cn
**Brazil**	www.enago.com.br, www.ulatus.com.br
**Germany**	www.enago.de
**Russia**	www.enago.ru
**Arabic**	www.enago.ae
**Turkey**	www.enago.com.tr
**S. Korea**	www.enago.co.kr
**Global**	www.enago.com, www.ulatus.com, www.voxtab.com

About Crimson:
Crimson Interactive INC is one of the world's leading academic research support services. Since 2005, we've supported over 2 million researchers in 125 countries with their publication goals.
